# Supplementary material for: Transcriptomic Analysis of Ficus carica Peels with a Focus on the Key Genes for Anthocyanin Biosynthesis
Source: Int J Mol Sci. 2020 Feb 13;21(4):1245. doi: 10.3390/ijms21041245 (PMC7072940; doi:10.3390/ijms21041245)
Supplement: Supplementary file 1 [file ijms-21-01245-s001.zip › Figure S1.docx]

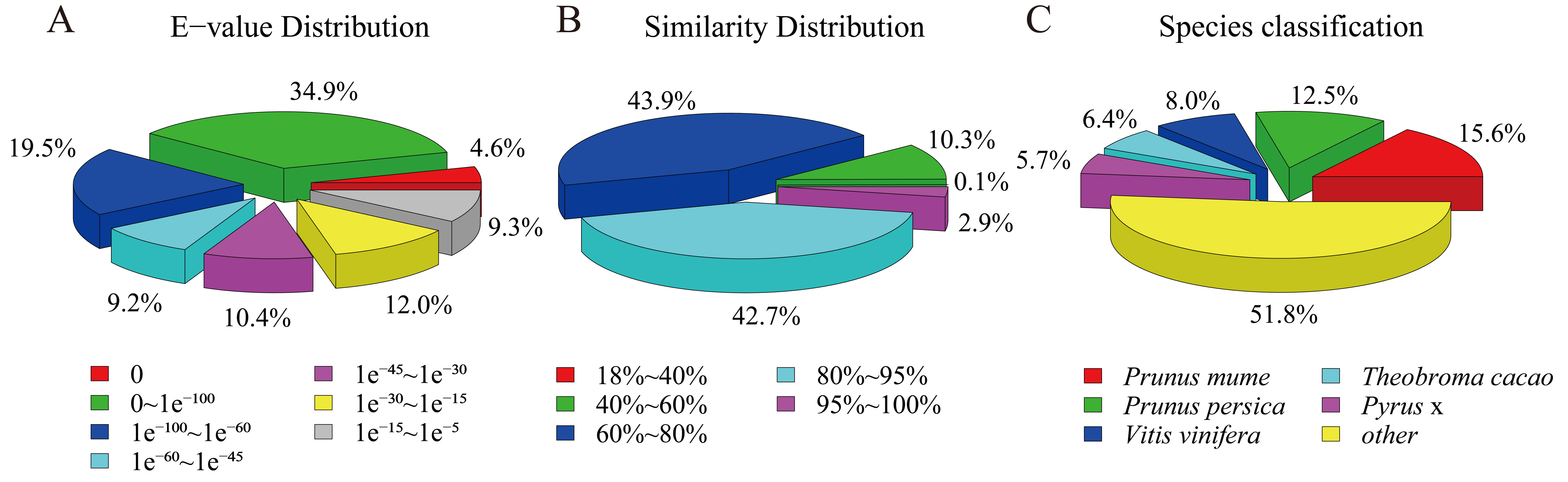


**Figure S1.** Annotation characteristics of fig peel unigenes in Nr database; (**A**) E-value distribution of Nr annotation; (**B**) Similarity distribution of Nr annotation; (**C**) Species distribution of Nr annotation. Nr, the NCBI non-redundant protein sequences.
