# Supplementary material for: Transcriptomic Analysis of Ficus carica Peels with a Focus on the Key Genes for Anthocyanin Biosynthesis
Source: Int J Mol Sci. 2020 Feb 13;21(4):1245. doi: 10.3390/ijms21041245 (PMC7072940; doi:10.3390/ijms21041245)
Supplement: Supplementary file 1 [file ijms-21-01245-s001.zip › Figure S2.docx]

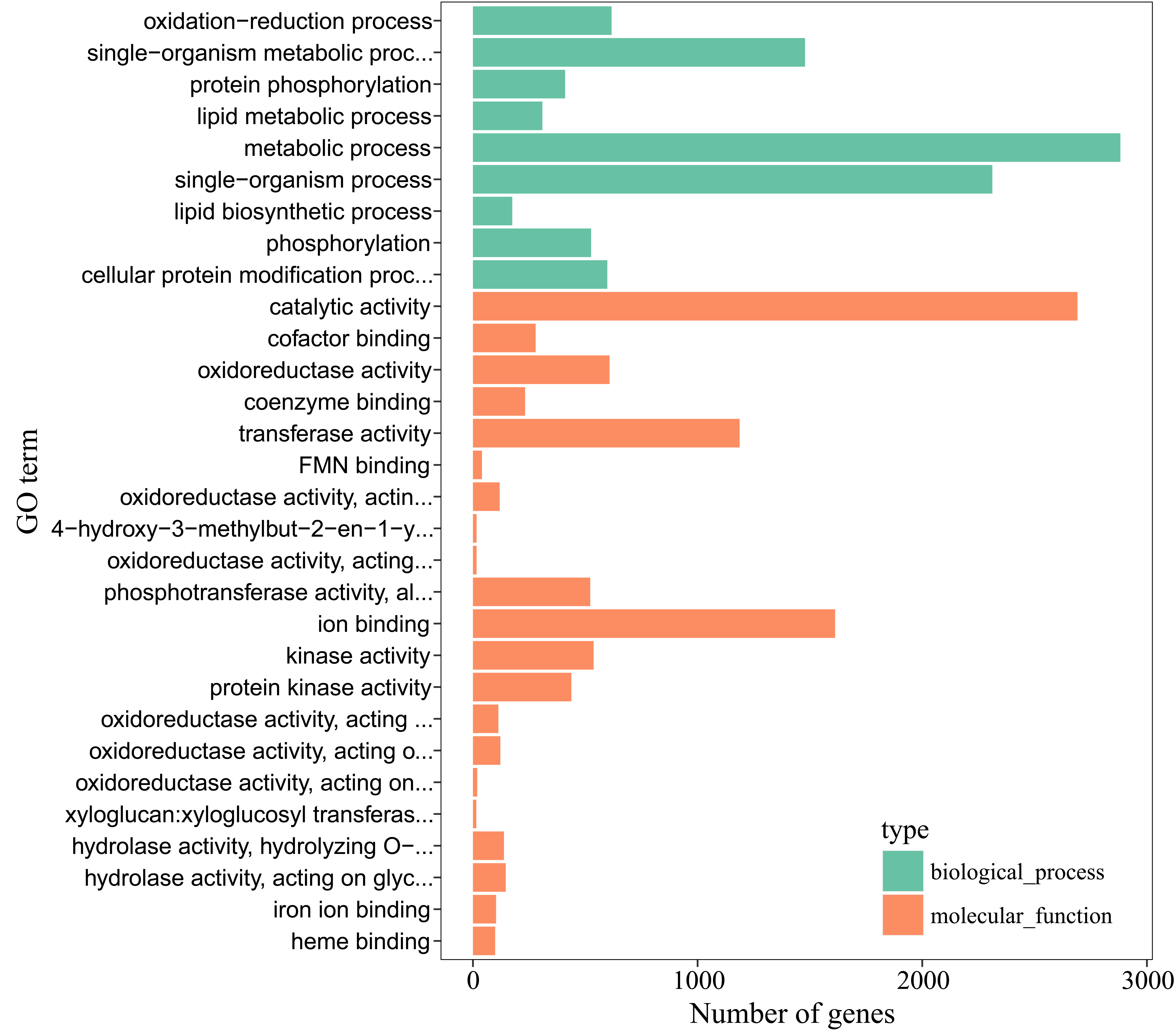


**Figure S2.** The top 30 GO enriched pathways of DEGs. GO, Gene Ontology; DEGs, differentially expressed genes.
