# Supplementary material for: Transcriptomic Analysis of Ficus carica Peels with a Focus on the Key Genes for Anthocyanin Biosynthesis
Source: Int J Mol Sci. 2020 Feb 13;21(4):1245. doi: 10.3390/ijms21041245 (PMC7072940; doi:10.3390/ijms21041245)
Supplement: Supplementary file 1 [file ijms-21-01245-s001.zip › Figure S3.docx]

**Figure S3.** qRT-PCR verify the validation of RNA-seq. Expression levels of the selected genes were measured by qRT-PCR (left Y axis) with the 2^-ΔΔCT^ method. *Fc18sRNA* was used as internal control, the line graphs represent the FPKM value (right Y axis) of DEGs from RNA-seq. Y and R represent yellow and red stages, respectively. Each data are means of three replicates. PAL, phenylalanine ammonium lyase, Cluster-11055.82174; C4H, cinnamic acid 4-hydroxylase, Cluster-11055.80529; CHS, chalcone synthase, Cluster-11055.81810; CHI, chalcone isomerase, Cluster-11055.10763; F3'H, flavanone 3'-hydroxylase, Cluster-11055.79806; DFR, dihydroflavonol-4-reductase, Cluster-11055.59175; ANS, anthocyanidin synthase, Cluster-11055.79498; UFGT, UDP-glucose: flavonoid 3-O-glucosyl transferase, Cluster-11055.59183; *FcMYB123*, Cluster-11055.86015; IAA, auxin-responsive protein, Cluster-11055.114052; PYL4*,* abscisic acid receptor PYL4, Cluster-11055.73430; ETR, ethylene-responsive transcription factor, Cluster-11055.67527. qRT-PCR, quantitative real-time PCR; RNA-seq, RNA-sequencing; DEGs, differentially expressed genes; FPKM, fragments per kilobase of gene per million mapped reads.
